# Membrane Protein Glycosylation Revisited: Functional Dynamics and Emerging Clinical Insights

**DOI:** 10.3390/ijms27083575

**Published:** 2026-04-16

**Authors:** Kyung-Hee Kim, Byong Chul Yoo

**Affiliations:** 1 Department of Applied Chemistry, School of Science and Technology, Kookmin University, Seoul 02707, Republic of Korea; kyungheekim@kookmin.ac.kr; 2 Antibody Research Institute, Kookmin University, Seoul 02707, Republic of Korea; 3 Diagnostic Research Team, InnoBation Bio R&D Center, Seoul 03929, Republic of Korea

**Keywords:** membrane proteins, glycosylation, N-linked glycans, glycoproteomics, clinical translation

## Abstract

Glycosylation is one of the most prevalent post-translational modifications of membrane proteins and plays a central role in regulating their structure and function. Unlike many existing reviews that address glycosylation in a system-wide context, this review focuses specifically on membrane proteins and examines how glycosylation shapes their functional behavior and clinical relevance. Because membrane proteins are exposed to the extracellular environment, glycans on their surface directly influence protein folding, receptor organization, and interactions with ligands and immune components. These diverse effects can be understood within a common mechanistic framework in which glycosylation modulates protein conformation, receptor clustering, and membrane organization, thereby altering signaling, adhesion, transport, and immune recognition. We discuss how N-linked and O-linked glycosylation regulate major classes of membrane proteins across these processes. Particular attention is given to disease-associated alterations in glycosylation, especially in cancer, immune and inflammatory disorders, and metabolic disease. For instance, glycosylation-dependent stabilization of PD-L1 and modulation of receptor signaling, such as EGFR, illustrate how glycan modifications contribute to immune evasion and therapeutic response. We further consider the clinical implications of membrane protein glycosylation, including its roles in biomarker development and as a potential target for therapeutic intervention. Advances in glycoproteomic technologies have enabled increasingly detailed characterization of site-specific glycosylation, although significant analytical challenges remain, particularly for membrane proteins. Overall, this review highlights membrane protein glycosylation as a dynamic regulatory layer that links molecular mechanisms to functional outcomes and clinical applications.

## 1. Introduction

Membrane proteins constitute approximately one-third of the human proteome and represent the majority of clinically approved drug targets, underscoring their central importance in physiology and disease [1,2]. These proteins mediate a wide range of biological processes, including signal transduction, molecular transport, cell–cell adhesion, and immune recognition, thereby functioning at the interface between intracellular regulatory networks and the extracellular environment [3]. Owing to their surface-exposed domains, membrane proteins are uniquely positioned to interact with soluble ligands, extracellular matrix components, and immune surveillance mechanisms, enabling cells to integrate environmental cues with intracellular signaling pathways [4]. However, despite this broad recognition, the specific implications of glycosylation for membrane protein function and clinical translation remain insufficiently integrated.

A defining molecular feature of many membrane proteins is their extensive glycosylation. Glycan moieties are predominantly attached to extracellular domains, where they contribute not only to protein folding and structural stability but also to functional regulation of receptor activity, ligand binding, and intermolecular interactions [5,6]. Although early studies regarded glycosylation primarily as a structural modification, accumulating evidence indicates that glycosylation is a highly regulated, dynamic, and context-dependent process that actively modulates membrane protein function and signaling behavior [7,8].

Membrane protein glycosylation exhibits remarkable structural diversity. Unlike proteins or nucleic acids, glycans are synthesized through non-template-driven enzymatic pathways involving coordinated activities of glycosyltransferases and glycosidases, resulting in heterogeneous glycoforms that vary according to cell type, developmental stage, metabolic status, and pathological condition [9,10]. This heterogeneity enables glycosylation to function as an integrative molecular layer that reflects both intrinsic genetic programs and extrinsic environmental influences [6,8]. In some contexts, alterations in membrane protein glycosylation have been reported to precede detectable changes in protein abundance or gene expression, although this may depend on cell type and pathological conditions [11].

Importantly, multiple forms of protein glycosylation exist (Table 1). In this review, we primarily focus on extracellular and luminal glycosylation of membrane proteins, particularly N-linked glycosylation and mucin-type O-glycosylation, which represent the predominant modifications affecting membrane protein folding, trafficking, and signaling. Other types of glycosylation, including O-GlcNAc modification, O-fucosylation, and C-mannosylation, are increasingly recognized as important regulatory processes but fall outside the main scope of this review, as they frequently occur in distinct intracellular contexts or specialized signaling pathways [12]. This distinction reflects the membrane topology constraint discussed above, as these modifications often occur in intracellular compartments rather than on extracellular domains of membrane proteins.

**Figure 1:**
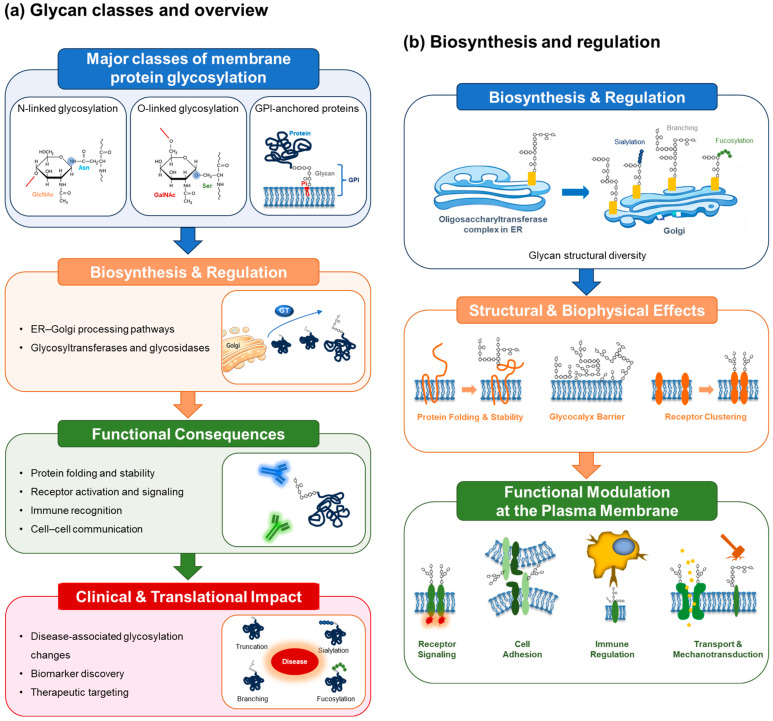

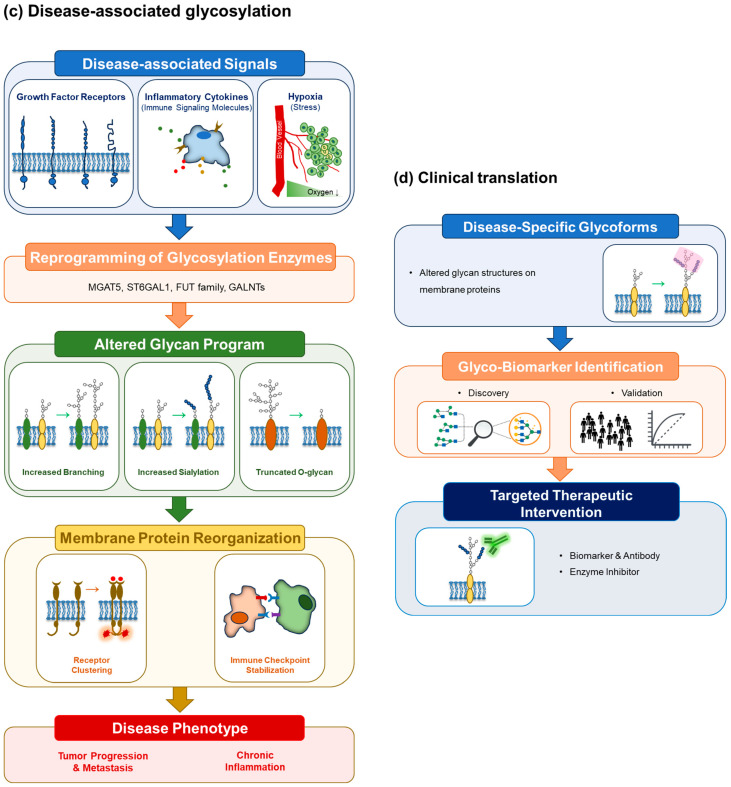

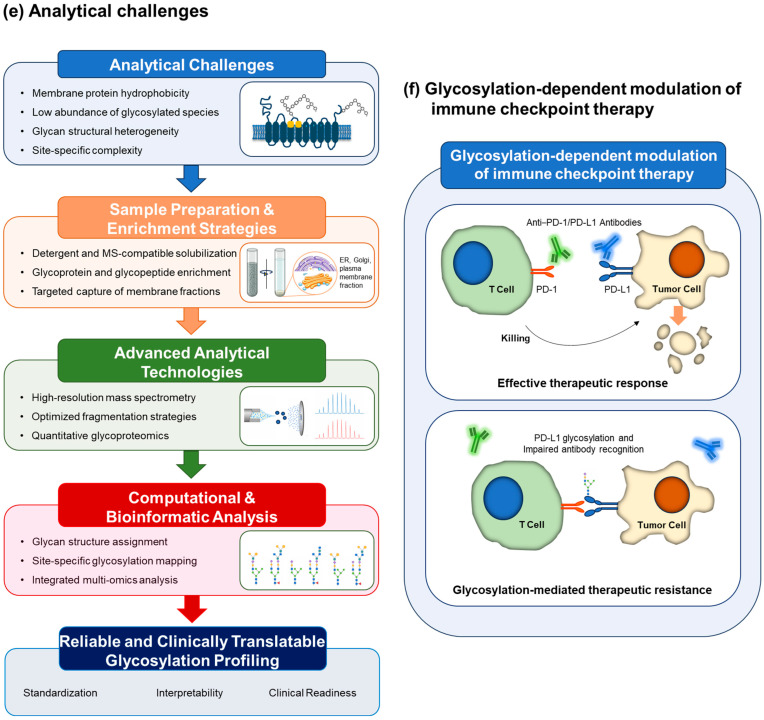
Integrated framework of membrane protein glycosylation: from biosynthesis and structural regulation to disease association, clinical translation, and analytical challenges. Membrane protein glycosylation represents a dynamic regulatory layer that connects intracellular biosynthetic pathways with membrane protein structure, cellular signaling, disease progression, and clinical applications. (**a**) Glycan classes and overview. Membrane proteins undergo diverse forms of glycosylation, including N-linked glycosylation, mucin-type O-linked glycosylation, and glycosylphosphatidylinositol (GPI) anchoring. These glycan modifications are generated through non-template-driven enzymatic pathways, producing structurally heterogeneous glycoforms that vary according to cell type, metabolic state, and physiological context. (**b**) Biosynthesis and regulation of membrane protein glycosylation. Glycosylation is initiated during protein biosynthesis within the endoplasmic reticulum (ER), where the oligosaccharyltransferase complex catalyzes N-linked glycan transfer. Subsequent processing in the Golgi apparatus involves sequential action of glycosyltransferases and glycosidases, generating structurally diverse glycan modifications such as branching, sialylation, and fucosylation. These processes influence membrane protein folding, stability, and structural organization, thereby shaping downstream functional behavior. (**c**) Disease-associated glycosylation reprogramming. In pathological conditions, disease-associated signals, including growth factor signaling, inflammatory cytokines, and hypoxia, reprogram glycosylation enzymes and glycan biosynthetic pathways. This rewiring results in characteristic alterations in membrane protein glycosylation, including increased N-glycan branching, enhanced sialylation, and truncated O-glycan structures. These changes promote receptor clustering, immune checkpoint stabilization, and membrane protein reorganization, contributing to disease phenotypes such as tumor progression and chronic inflammation. (**d**) Clinical translation and therapeutic targeting. Disease-specific glycoforms of membrane proteins provide opportunities for biomarker discovery, patient stratification, and therapeutic intervention. Glycosylation-dependent biomarkers can be detected through glyco-diagnostics and antibody-based assays, while therapeutic strategies include glyco-engineered antibodies and pharmacological inhibition of glycosyltransferases or related pathways. (**e**) Analytical challenges and emerging technologies. Comprehensive analysis of membrane protein glycosylation remains technically challenging due to membrane protein hydrophobicity, glycan heterogeneity, and site-specific complexity. Advances in glycoproteomics, including improved enrichment strategies, high-resolution mass spectrometry, and computational analysis, are enabling more accurate site-specific glycosylation mapping and integration with multi-omics datasets, thereby supporting translational and clinical applications. (**f**) Glycosylation-dependent modulation of immune checkpoint therapy. In the absence of glycosylation-mediated interference, anti–PD-1/PD-L1 antibodies effectively block receptor–ligand interactions, restoring T cell-mediated cytotoxicity against tumor cells. In contrast, glycosylation of immune checkpoint molecules, such as PD-L1, can impair antibody recognition and binding, thereby reducing therapeutic efficacy and contributing to glycosylation-mediated therapeutic resistance. Collectively, these panels summarize the key mechanistic principles linking glycosylation-dependent conformational regulation, receptor clustering, and membrane organization to functional outcomes and clinical applications.

Aberrant glycosylation of membrane proteins has emerged as a recurrent feature of human disease. In cancer, membrane proteins frequently display increased branching of N-linked glycans, truncation of mucin-type O-glycans, and altered terminal modifications such as sialylation and fucosylation [7,8]. These changes can modulate receptor signaling, cell adhesion, migration, and immune evasion, thereby contributing to tumor progression and metastasis [13,14,15]. Similar principles apply to immune and inflammatory disorders, in which dysregulated glycosylation of membrane receptors and adhesion molecules alters immune cell activation thresholds, trafficking, and effector functions [16,17]. The conceptual framework of this review, linking glycosylation biosynthesis, structural regulation, disease-associated reprogramming, and clinical translation, is summarized in Figure 1 and developed further in the following sections.

Despite its functional significance, membrane protein glycosylation is often discussed only tangentially within the broader glycosylation literature. Many existing reviews adopt a system-wide perspective on glycosylation or focus predominantly on soluble glycoproteins and secreted biomarkers, often without fully addressing how glycosylation specifically modulates membrane protein behavior at the cell surface [10,11]. In contrast, a membrane protein-centric framework that explicitly connects glycosylation-dependent molecular mechanisms with clinically relevant phenotypes remains relatively underdeveloped. This represents a notable gap, given that membrane proteins constitute the principal class of diagnostic markers and therapeutic targets across multiple disease areas [2,18].

From a mechanistic standpoint, glycosylation influences membrane protein biology at multiple hierarchical levels. During biosynthesis, N-linked glycosylation governs protein folding and quality control within the endoplasmic reticulum, thereby determining whether a membrane protein is correctly trafficked to the cell surface or targeted for degradation [5,19]. At the plasma membrane, glycan structures can influence receptor conformation, lateral mobility, and clustering within membrane microdomains, ultimately affecting ligand binding kinetics and downstream signaling strength [20,21]. In addition, membrane protein-associated glycans may serve as ligands for endogenous lectins, enabling glycosylation-dependent communication between cells, particularly within immune systems and inflammatory microenvironments [7,16,22].

The clinical relevance of these molecular effects is increasingly evident. Glycosylation-dependent modulation of membrane protein signaling has been implicated in therapeutic resistance, variable drug response, and disease progression across oncology, immunology, and metabolic medicine [8,13]. Importantly, disease-associated glycoforms of membrane proteins can often be detected in circulating extracellular vesicles or shed ectodomains, creating opportunities for minimally invasive biomarker development [23,24].

Technological advances have further accelerated interest in membrane protein glycosylation. Improvements in mass spectrometry-based glycoproteomics, glycopeptide enrichment strategies, and computational analysis now permit site-specific and structure-resolved characterization of glycosylation, even within complex membrane proteomes [25,26,27]. Nevertheless, membrane proteins remain analytically challenging due to their hydrophobicity, low abundance, and glycan heterogeneity [28,29].

In parallel with these advances, emerging structural and computational technologies are beginning to further transform the analysis of membrane protein glycosylation. Recent developments in artificial intelligence-based protein structure prediction, exemplified by AlphaFold, have enabled increasingly accurate modeling of membrane protein architectures, providing a structural framework for interpreting the spatial context of glycosylation sites [30]. These advances are particularly valuable for visualizing glycosylation-dependent structural heterogeneity (glycoforms) in membrane proteins. In addition, high-resolution structural techniques such as cryo-electron microscopy (cryo-EM) are allowing direct visualization of glycosylated membrane protein complexes, offering new insights into glycan-dependent conformational regulation and receptor organization [31]. Furthermore, spatially resolved glycomics approaches are emerging as powerful tools to map glycan distributions within tissue contexts, enabling the integration of glycosylation patterns with cellular microenvironments [32]. Together, these technologies are bridging the gap between glycoproteomic profiling and structural–functional interpretation, thereby strengthening the mechanistic understanding of membrane protein glycosylation.

In this review, we focus on glycosylation of membrane proteins and examine how molecular glycosylation mechanisms translate into clinically meaningful outcomes. By integrating insights from glycobiology, membrane protein biochemistry, and clinical research, we discuss biosynthesis, functional consequences, disease associations, and translational opportunities. Through this membrane protein-centric perspective, we aim to highlight glycosylation as an important regulatory dimension of membrane biology and a potentially actionable layer of disease-associated molecular regulation.

**Table 1:** Major classes of protein glycosylation relevant to membrane protein biology and their biological characteristics.

Glycosylation Type	Biosynthetic Location	Glycan Modification	Major Enzymes	Structural Features	Representative Membrane Proteins	Functional Consequences	Key Reference
N-linked glycosylation	ER and Golgi	Core glycan attachment in the ER followed by trimming and branching in the Golgi	Oligosaccharyltransferase, glycosidases, glycosyltransferases	Attachment of preassembled oligosaccharides to Asn residues (Asn–X–Ser/Thr sequon)	EGFR, integrins, ion channels	Protein folding, receptor stability, ligand binding, signaling regulation	[5,19]
Mucin-type O-linked glycosylation	Golgi apparatus	Initiation by GalNAc addition followed by elongation into diverse O-glycan structures	Polypeptide GalNAc-transferases	Addition of GalNAc to Ser/Thr residues, followed by elongation into diverse O-glycan structures	MUC1, adhesion receptvors	Extracellular domain extension, steric regulation, cell–cell interactions	[12,33]
O-GlcNAcylation *	Cytoplasm and nucleus	Dynamic addition and removal of single GlcNAc residues	O-GlcNAc transferase (OGT)	Addition of single GlcNAc to Ser/Thr residues	Signaling-associated membrane proteins **	Signal regulation and metabolic sensing	[12]
O-fucosylation	ER and Golgi	Addition and processing of fucose-containing glycans on specific protein motifs	Protein O-fucosyltransferases	Addition of fucose residues to specific protein motifs	Notch receptors	Regulation of receptor–ligand interactions and signaling	[12]
O-mannosylation	ER and Golgi	Addition and extension of mannose-containing O-glycans	Protein O-mannosyltransferases	Mannose addition to Ser/Thr residues, often followed by further elongation	α-Dystroglycan, cadherins	Protein stability, receptor function, cell–matrix interactions	[12]
C-mannosylation	ER	Mannose addition to tryptophan residues	C-mannosyltransferases	Mannose addition to tryptophan residues	Secreted and membrane signaling proteins	Protein folding and stability	[12]
GPI-anchored proteins	ER (anchor attachment), Golgi (remodeling)	GPI anchor attachment followed by glycan and lipid remodeling	GPI transamidase complex, remodeling enzymes	Post-translational attachment of a glycosylphosphatidylinositol anchor to the C-terminus	CD55, CD59, alkaline phosphatase	Membrane anchoring, signal modulation, immune recognition	[12]

* Mentioned for conceptual completeness but not discussed in detail in this review. ** Examples include signaling-associated membrane proteins and receptors undergoing intracellular trafficking.

## 2. Biosynthesis and Regulation of Membrane Protein Glycosylation

The biosynthesis of membrane protein glycosylation is a tightly coordinated, multistep process that integrates protein translation, intracellular trafficking, and enzymatic modification within the secretory pathway. In contrast to many cytosolic post-translational modifications, most glycosylation events affecting membrane proteins occur within the lumen of the endoplasmic reticulum (ER) and Golgi apparatus. This spatial restriction ensures that glycan moieties are selectively displayed on luminal or extracellular domains of membrane proteins following trafficking to the cell surface [5]. Such topological organization is a defining feature of membrane protein glycosylation and contributes directly to its functional specificity in extracellular signaling and cell–cell communication. Together, glycosylation across the ER and Golgi constitutes a coordinated process in which early folding and quality control are coupled to later structural diversification. These sequential steps ultimately shape the surface organization, stability, and functional behavior of membrane proteins. This framework—centered on conformational regulation, receptor clustering, and membrane organization—provides a basis for organizing the following sections, which examine glycosylation across biosynthetic processes, structural effects, functional modulation, and disease contexts.

### 2.1. Co-Translational N-Linked Glycosylation in the Endoplasmic Reticulum

N-linked glycosylation of membrane proteins is typically initiated co-translationally in the ER as nascent polypeptide chains are translocated into the ER lumen through the Sec61 translocon [5,34]. The oligosaccharyltransferase (OST) complex catalyzes the en bloc transfer of a preassembled oligosaccharide to asparagine residues within the consensus sequon Asn–X–Ser/Thr. For membrane proteins, the accessibility of this sequon is strongly influenced by membrane topology, luminal loop length, and transmembrane domain orientation [29,35].

This early glycosylation step plays a central role in protein folding and quality control. N-glycans function as molecular tags for lectin chaperones such as calnexin and calreticulin, which assist in proper folding and prevent premature export of misfolded membrane proteins [5,19]. Failure to acquire or correctly process N-glycans frequently results in ER retention and subsequent degradation through the ER-associated degradation (ERAD) pathway, a mechanism implicated in both inherited glycosylation disorders and acquired disease states [36,37].

Importantly, not all potential N-glycosylation sites are necessarily occupied. Site occupancy is influenced by local sequence context, translation kinetics, and cellular metabolic conditions, introducing an additional regulatory layer that can modulate membrane protein function without altering amino acid sequence [29,38,39]. Recent glycoproteomic analyses further indicate that site-specific N-glycosylation patterns can vary substantially across tissues and disease contexts [25,26]. This variability in site occupancy may contribute to functional heterogeneity of membrane proteins, as differences in glycan occupancy can influence receptor stability, surface expression, and signaling responsiveness.

### 2.2. Golgi Processing and Structural Diversification of N-Glycans

Following ER exit, glycosylated membrane proteins traverse the Golgi apparatus, where N-glycans undergo extensive trimming and remodeling. Sequential actions of glycosidases and glycosyltransferases generate high-mannose, hybrid, or complex N-glycan structures [9,40]. This maturation process is strongly influenced by Golgi organization, enzyme localization, and substrate availability, producing cell type-specific glycosylation patterns.

For membrane proteins, Golgi-mediated glycan remodeling can have direct functional consequences. Increased branching of complex N-glycans promotes interactions with galectins and stabilizes receptor confinement within membrane microdomains, thereby influencing receptor residence time and signaling amplitude [20,21]. Conversely, alterations in terminal modifications such as sialylation or fucosylation can modulate receptor–ligand interactions, immune recognition, and receptor turnover.

Golgi processing is also dynamically regulated by cellular conditions including hypoxia, nutrient availability, and inflammatory signaling. These inputs link metabolic and environmental cues to membrane protein glycosylation states, thereby coupling glycan remodeling to broader cellular regulatory networks [8,21]. This process is further influenced by the spatial organization of Golgi enzymes, where cisternal compartmentalization and enzyme localization can regulate the sequential processing and structural diversification of glycans. In addition to biosynthetic regulation, glycan remodeling can also occur at the cell surface through the action of neuraminidases, which remove terminal sialic acid residues. This desialylation can alter receptor charge, ligand interactions, and immune recognition, thereby dynamically modulating membrane protein function [41,42].

### 2.3. O-Linked Glycosylation and Golgi-Based Regulation

In contrast to N-linked glycosylation, mucin-type O-linked glycosylation is generally initiated in the Golgi apparatus through the transfer of N-acetylgalactosamine (GalNAc) to serine or threonine residues by members of the polypeptide GalNAc-transferase family [12,33]. The absence of a strict consensus sequence allows extensive heterogeneity and context-dependent regulation of O-glycosylation.

Membrane proteins enriched in O-glycans often possess extended extracellular domains that influence receptor accessibility, protease susceptibility, and mechanical properties at the cell surface [43,44]. In several disease contexts, particularly cancer, dysregulated O-glycosylation results in the accumulation of truncated glycan structures such as Tn or sialyl-Tn antigens, which can alter receptor signaling, immune recognition, and cell adhesion [45].

It should be noted that multiple forms of O-glycosylation exist beyond mucin-type glycosylation. Modifications such as O-GlcNAcylation, O-fucosylation, and O-mannosylation play important regulatory roles in specific cellular contexts. However, because many of these modifications occur within intracellular compartments or specialized signaling pathways, they are not discussed in detail in the present membrane protein-focused framework [12]. Consistent with the mechanistic framework described above, these alterations can be understood within the broader regulatory context in which glycosylation modulates protein conformation, receptor accessibility, and membrane organization, thereby contributing to disease-associated phenotypes.

### 2.4. Regulation by Cellular Context and Membrane Topology

A distinctive feature of membrane protein glycosylation is its dependence on membrane topology. Only luminal and extracellular domains are accessible to the glycosylation machinery, making transmembrane orientation a key determinant of glycan distribution [29,35]. Changes in membrane protein conformation, trafficking dynamics, or membrane organization can therefore indirectly influence glycosylation patterns.

Cell type specificity further contributes to glycosylation diversity. Differential expression of glycosyltransferases, nucleotide sugar transporters, and Golgi-resident enzymes generates characteristic glycosylation signatures across tissues and disease states [9,12]. Advances in glycoproteomics have revealed that such cell-specific glycosylation programs are highly dynamic and responsive to physiological perturbations [12,25].

### 2.5. Integration of Glycosylation with Disease-Associated Signaling Pathways

Perturbations in glycosylation biosynthesis are increasingly recognized as tightly linked to disease-associated signaling networks. For example, oncogenic pathways such as PI3K–AKT and MAPK signaling have been linked to the regulation of glycosyltransferase expression and glycan remodeling in cancer cells [8,20]. In this context, rather than representing merely downstream consequences of pathology, alterations in glycosylation machinery can arise from signaling pathways that regulate glycosyltransferase expression, nucleotide sugar metabolism, and Golgi organization.

In cancer, oncogenic signaling pathways have been shown to transcriptionally and post-transcriptionally reprogram glycosylation enzymes, including branching-associated N-acetylglucosaminyltransferases and terminal sialyltransferases [8,20]. Such regulatory shifts modify glycan processing capacity within the ER–Golgi network, thereby altering the structural landscape of membrane protein glycosylation.

Similarly, inflammatory cytokines and metabolic stress signals influence glycosylation by modulating glycosyltransferase activity and substrate availability [16,45]. Changes in nutrient flux through the hexosamine biosynthetic pathway can further reshape glycan branching potential, linking cellular metabolic state to membrane protein glycosylation patterns [6].

Collectively, these regulatory mechanisms operate at the level of biosynthetic control and position glycosylation as a responsive and tunable modification integrated with broader cellular signaling programs. The downstream phenotypic consequences of such glycosylation reprogramming are discussed in Section 5. The key regulatory steps in membrane protein glycosylation biosynthesis are summarized in Table 2.

## 3. Structural and Biophysical Consequences of Membrane Protein Glycosylation

Glycosylation exerts profound effects on the structural and biophysical properties of membrane proteins, extending far beyond its canonical role in biosynthetic quality control. In this context, these structural effects can be interpreted within the mechanistic framework described above, particularly in terms of conformational regulation and membrane organization. By adding bulky, hydrophilic, and conformationally flexible moieties to extracellular domains, glycosylation reshapes protein folding landscapes, stabilizes specific conformational states, and modulates protein dynamics at the cell surface [7,47]. Increasing structural and glycoproteomic evidence indicates that glycan composition can influence receptor architecture, intermolecular interactions, and membrane organization, thereby linking glycosylation states to functional signaling outputs. Such effects may arise both from direct glycan–protein interactions, such as steric stabilization or shielding of hydrophobic regions, and from indirect solvent-mediated influences on hydration and conformational flexibility [47,48].

### 3.1. Glycosylation-Dependent Folding and Conformational Stability

The contribution of glycosylation to membrane protein folding begins during biosynthesis but continues to influence conformational stability after surface expression. N-linked glycans can stabilize folded domains by reducing local conformational entropy and shielding hydrophobic patches from solvent exposure [47,48]. For membrane proteins with large extracellular domains, including receptor tyrosine kinases and adhesion molecules, glycosylation frequently defines the boundaries of folded domains and interdomain flexibility.

Experimental and computational studies have demonstrated that removal or alteration of specific N-glycosylation sites can destabilize native conformations, increase susceptibility to proteolysis, and promote misfolding or aggregation [5]. For example, site-specific N-glycosylation has been shown to regulate the folding and stability of receptors such as the epidermal growth factor receptor (EGFR) and other receptor tyrosine kinases [20]. In particular, site-specific N-glycosylation can influence EGFR dimerization and signaling amplitude, thereby fine-tuning receptor activation dynamics. These effects are particularly relevant for receptors whose activation depends on precise conformational transitions, as glycosylation can bias the equilibrium toward inactive or active states [49,50].

### 3.2. Regulation of Extracellular Domain Architecture

Beyond local stability, glycosylation also influences the global architecture of extracellular domains. O-linked glycosylation, particularly in mucin-like regions, introduces extended and semi-rigid structures that increase the effective size and spatial reach of membrane proteins [33,43]. This architectural effect can sterically modulate ligand access, receptor dimerization, and interactions with neighboring membrane proteins.

For membrane proteins involved in cell–cell adhesion and immune recognition, glycan-mediated spacing effects are especially important. For example, dense glycosylation of mucin-like proteins such as MUC1 contributes to the formation of an expanded glycocalyx layer that can alter receptor accessibility and modulate signaling thresholds [44]. Similarly, glycosylation of adhesion receptors such as integrins can influence receptor–ligand engagement and cell–matrix interactions [14].

Dense glycosylation at the cell surface can therefore create a protective glycocalyx that physically separates the plasma membrane from extracellular ligands, altering signaling thresholds and mechanical sensitivity [44,51]. These principles are illustrated in Figure 1b, which summarizes how glycosylation shapes extracellular domain organization and surface presentation. Glycan-dependent spacing may also influence receptor competition and spatial exclusion at the cell surface, potentially altering ligand accessibility and signaling hierarchies [44,51].

### 3.3. Influence on Lateral Mobility and Membrane Microdomains

At the plasma membrane, glycosylation affects not only protein structure but also biophysical behavior within the lipid bilayer. Glycan–lectin interactions, particularly involving galectins, can promote receptor clustering and confinement within specific membrane regions [20,21]. This phenomenon is frequently described as the formation of a galectin–glycoprotein lattice that regulates receptor surface retention and signaling capacity [20,21].

Such clustering can enhance or prolong signaling by stabilizing receptor complexes and reducing lateral diffusion. For instance, galectin-mediated interactions with branched N-glycans have been reported to influence the membrane organization of receptors such as EGFR and T-cell receptors [20,21].

Conversely, alterations in glycan composition can disrupt these interactions, increasing receptor mobility and attenuating downstream signaling [52]. Glycosylation-dependent organization of membrane proteins into specialized membrane microdomains, including lipid rafts, can further influence receptor clustering, accessibility, and signal transduction efficiency [21,45]. The concept of lipid rafts remains subject to ongoing debate, and their definition and functional relevance may vary depending on experimental context [21,45].

### 3.4. Mechanical and Biophysical Implications

Membrane protein glycosylation also contributes to the mechanical properties of the cell surface. Glycans form hydrated and highly flexible layers that increase steric repulsion and generate a viscoelastic barrier at the cell surface [44,51]. These glycan-rich structures influence how cells respond to compressive forces, shear stress, and mechanical deformation. Mechanical effects at the cell surface can be interpreted as an extension of glycan-induced structural organization, where increased spacing and hydration translate into altered biophysical properties [44,51].

For mechanosensitive receptors and adhesion molecules, glycosylation can modulate force transmission and signal initiation. Changes in glycosylation patterns have been shown to alter cellular stiffness, adhesion strength, and migratory behavior, particularly in cancer cells and activated immune cells [44]. These observations link glycan remodeling to mechanical phenotypes such as increased invasiveness and altered immune cell trafficking.

### 3.5. Structural Heterogeneity and Functional Plasticity

Building on these structural and biophysical considerations, a defining feature of glycosylation is its inherent heterogeneity, which introduces structural plasticity at the membrane protein level. Different glycoforms of the same membrane protein can coexist on the cell surface, each with distinct conformational and biophysical properties [9,53]. Advances in site-specific glycoproteomics have further revealed that such glycoform diversity can dynamically change in response to metabolic state, cellular signaling, and disease progression [25,26].

Glycoform heterogeneity also presents challenges for experimental reproducibility and structural analysis, as different glycoforms may not be equally represented or resolved in biochemical and structural studies [25,26]. From a clinical perspective, glycoform-specific structural differences can also influence therapeutic efficacy, as drugs and antibodies may preferentially recognize particular glycosylation states of target proteins such as PD-L1 or receptor tyrosine kinases [15].

Collectively, these observations highlight the importance of integrating structural glycosylation analysis into drug development and precision medicine strategies. The major structural and biophysical consequences of membrane protein glycosylation are summarized in Table 3.

## 4. Functional Modulation of Membrane Proteins by Glycosylation

Glycosylation is a central determinant of membrane protein function, acting as a dynamic regulator of receptor signaling, molecular transport, cell–cell adhesion, and immune recognition. These functional consequences can be understood as downstream manifestations of glycosylation-induced changes in conformation, receptor clustering, and membrane organization. By modulating protein conformation, surface organization, and intermolecular interactions, glycosylation fine-tunes functional outputs without altering the underlying amino acid sequence [6,7,21]. This section focuses on how glycosylation shapes the functional behavior of major classes of membrane proteins and how these effects translate into clinically relevant phenotypes.

### 4.1. Regulation of Receptor Signaling

Receptor-mediated signaling represents one of the most extensively studied functional consequences of membrane protein glycosylation. Glycosylation can influence receptor activity at multiple levels, including ligand binding affinity, receptor dimerization, activation kinetics, and signal duration [6,7]. For many receptors, particularly receptor tyrosine kinases and G protein-coupled receptors, specific glycosylation sites are essential for proper receptor maturation and surface expression [5,50]. These effects can be broadly distinguished into those that influence ligand binding and receptor activation at the cell surface, and those that modulate downstream signaling dynamics, including signal duration and amplification [6,7,20].

For example, N-glycosylation of the epidermal growth factor receptor (EGFR) has been shown to regulate receptor folding, trafficking to the plasma membrane, and ligand-induced activation [20]. Similarly, glycosylation of immune checkpoint receptors such as programmed death-ligand 1 (PD-L1) can stabilize receptor expression at the cell surface and influence immune recognition [54]. This stabilization can prolong PD-L1 surface retention and enhance immune checkpoint signaling, thereby contributing to immune evasion and reduced therapeutic responsiveness.

To further strengthen the clinical translation of glycosylation-dependent targeting strategies, representative examples of approved and emerging therapeutic approaches are summarized in Table 4. Collectively, these observations highlight how glycosylation-dependent structural and functional modulation of membrane proteins can be directly translated into clinically actionable therapeutic strategies.

Beyond biosynthetic roles, glycosylation actively modulates signaling dynamics at the plasma membrane. Complex N-glycan branching can enhance interactions with galectins and promote receptor surface persistence and signaling amplitude [20,21]. This galectin-mediated lattice effect increases local receptor density and stabilizes signaling complexes, resulting in amplified and sustained downstream signaling [20,21]. Conversely, loss or simplification of glycan structures can increase receptor internalization and attenuate signaling responses [52]. These mechanisms are summarized in Figure 1b, which illustrates how glycosylation-dependent receptor organization shapes signaling outcomes.

### 4.2. Control of Cell–Cell and Cell–Matrix Adhesion

Cell adhesion molecules are heavily glycosylated membrane proteins whose functions are intrinsically linked to glycan-mediated regulation. Glycosylation of adhesion receptors influences binding strength, specificity, and mechanical stability of cell–cell and cell–matrix interactions [7,8]. In particular, N- and O-linked glycans modulate the spatial arrangement and flexibility of extracellular domains, thereby altering adhesion kinetics and force transmission.

Integrins represent a well-studied example of glycosylation-regulated adhesion receptors. Site-specific N-glycosylation of integrin subunits can influence receptor conformation, ligand binding affinity, and downstream signaling pathways [14]. Similarly, glycosylation of cadherins and other adhesion molecules contributes to the regulation of cell–cell junction stability and tissue architecture [14]. These effects are closely related to the mechanical properties of the cell surface described above, where glycan-mediated spacing and hydration influence adhesion strength, force transmission, and cellular responsiveness to mechanical cues [44,51].

Altered glycosylation of adhesion molecules has been implicated in pathological processes such as tumor invasion and metastasis. Increased sialylation or altered fucosylation patterns can reduce cell–cell adhesion while enhancing interactions with extracellular matrix components, facilitating cell migration [57,58]. These glycosylation-dependent changes in adhesion behavior provide a mechanistic link between molecular modification and tissue-level pathology [8].

### 4.3. Modulation of Transporters and Channels

Membrane transporters and ion channels represent another functional class profoundly influenced by glycosylation. Glycans can regulate transporter folding, membrane localization, and turnover, thereby affecting substrate flux across the membrane [59,60].

For example, glycosylation of glucose transporters such as GLUT1 has been shown to influence membrane localization and stability, thereby affecting cellular glucose uptake [6]. Similarly, glycosylation of ion channels, including voltage-gated sodium channels and CFTR, can modulate channel gating, trafficking, and degradation [29].

In some cases, glycosylation directly influences channel gating or transporter activity by stabilizing specific conformational states [61,62]. Functional alterations in transporter glycosylation have also been associated with metabolic disorders and drug resistance. Changes in glycosylation can modify transporter affinity for substrates or inhibitors, which can lead to variability in drug absorption, distribution, and therapeutic efficacy [11]. In addition, glycosylation-dependent changes in transporter stability and surface residence time may contribute to variability in substrate uptake and pharmacological response [11,59]. These observations underscore the importance of glycosylation in pharmacological responses.

### 4.4. Immune Receptors and Glycan-Mediated Recognition

Immune receptors are particularly sensitive to glycosylation-dependent regulation because glycan structures serve as recognition elements for endogenous lectins and immune modulators [7,16]. Glycosylation of membrane immune receptors can influence receptor clustering, signaling thresholds, and interactions with ligands or co-receptors.

For example, glycosylation of immune checkpoint molecules such as PD-L1 has been reported to regulate receptor stability and immune evasion in tumor cells [54]. In addition, interactions between sialylated glycans on membrane proteins and inhibitory Siglec receptors on immune cells can suppress immune activation and contribute to immune tolerance [16]. Depending on the glycan context, these interactions can either promote immune activation, for example, through enhanced receptor clustering, or mediate inhibitory signaling via engagement of lectin receptors such as Siglecs [16,45]. Diet-derived non-human glycans, such as α-Gal and Neu5Gc, can also be incorporated into membrane glycoproteins and elicit immune responses. These glycans have been implicated in inflammatory conditions and may influence immune recognition and antibody-mediated responses in humans [63,64].

Conversely, loss or modification of specific glycan structures can enhance immune activation and inflammatory signaling [16,45]. These mechanisms highlight glycosylation as a critical modulator of immune homeostasis and immune surveillance.

### 4.5. Functional Heterogeneity and Context-Dependent Regulation

A key feature of membrane protein glycosylation is its capacity to generate functional heterogeneity. Different glycoforms of the same protein can exhibit distinct functional behaviors, enabling context-dependent regulation of cellular responses [9,11]. Advances in glycoproteomic technologies have further revealed that membrane proteins frequently exist as heterogeneous populations of glycoforms with distinct functional properties [25,26].

This heterogeneity allows cells to adapt rapidly to environmental cues, such as changes in nutrient availability, inflammatory signaling, or therapeutic pressure. From a clinical perspective, glycosylation-driven functional heterogeneity can complicate biomarker interpretation and therapeutic targeting. Drugs or antibodies may preferentially recognize specific glycoforms, leading to variable efficacy across patient populations [49,55]. Such functional heterogeneity may also underlie variability in clinical outcomes, including differences in biomarker performance and therapeutic response [49,55].

Recognizing and characterizing this functional diversity is therefore essential for precision medicine. The principal functional roles of membrane protein glycosylation across major protein classes are summarized in Table 5.

## 5. Disease-Associated Alterations in Membrane Protein Glycosylation

Altered glycosylation of membrane proteins is increasingly recognized as a defining molecular feature of diverse human diseases. Unlike genetic mutations, which are relatively static, glycosylation changes are dynamic and quantitatively modulated, reflecting alterations in cellular metabolism, signaling pathways, and microenvironmental conditions [6,8,11]. Consequently, membrane protein glycosylation can function both as a driver of pathological processes and as a sensitive indicator of disease-associated cellular reprogramming [8,11,15].

This section examines disease-specific patterns of membrane protein glycosylation, with particular emphasis on cancer, followed by immune and inflammatory disorders and metabolic diseases. The major pathological glycosylation alterations and their functional consequences are summarized in Figure 1c and Table 6.

### 5.1. Cancer-Associated Glycosylation of Membrane Proteins

Cancer represents the most extensively studied context in which membrane protein glycosylation is dysregulated. Tumor cells frequently undergo substantial remodeling of glycosylation pathways, resulting in characteristic alterations in glycan structures displayed on membrane proteins [8,13]. Importantly, these changes are increasingly understood to actively contribute to malignant phenotypes, including sustained proliferative signaling, invasion, metastasis, and immune evasion [8,65].

One prominent feature of cancer-associated glycosylation is increased branching of complex N-linked glycans on membrane receptors. Enhanced activity of glycosyltransferases such as MGAT5 promotes β1,6-N-acetylglucosamine branching, which can strengthen galectin-mediated receptor interactions and prolong receptor residence at the cell surface [20,21]. Through the formation of a galectin–glycoprotein lattice, these branched glycans stabilize receptor clustering and amplify oncogenic signaling pathways [20,21]. This mechanism has been reported to influence signaling of growth factor receptors such as EGFR and other receptor tyrosine kinases [18] and may contribute to resistance to targeted therapies [66,67].

Cancer cells also frequently exhibit alterations in mucin-type O-glycosylation. Dysregulated expression of glycosyltransferases can lead to accumulation of truncated O-glycan structures, including Tn and sialyl-Tn antigens, on membrane proteins [68,69]. These truncated glycans can alter extracellular domain organization, reduce cell–cell adhesion, and expose peptide epitopes that facilitate tumor invasion and dissemination [45].

In addition, cancer cells often display increased terminal sialylation and altered fucosylation patterns on membrane proteins. Upregulation of sialyltransferases such as ST6GAL1 or fucosyltransferases such as FUT8 has been associated with enhanced receptor signaling, altered immune recognition, and metastatic potential [14]. These terminal glycan modifications can also influence interactions with lectin receptors on immune cells, thereby contributing to tumor immune evasion.

Together, these changes lead to recurring functional consequences, including enhanced receptor signaling, reduced cell–cell adhesion, and altered immune recognition [8,13,14,15].

### 5.2. Immune and Inflammatory Disorders

In immune and inflammatory diseases, altered glycosylation of membrane proteins can reshape immune cell behavior and tissue responses. Immune receptors, adhesion molecules, and cytokine receptors are heavily glycosylated, and changes in their glycosylation status can influence activation thresholds, receptor clustering, and downstream signaling outputs [6,16]. Such mechanisms have been implicated in autoimmune diseases and chronic inflammatory conditions, where altered glycosylation affects immune cell activation and tissue-specific responses [16,17].

Chronic inflammatory conditions are frequently associated with changes in sialylation and fucosylation of membrane proteins, which modulate interactions with lectin receptors expressed by immune cells [7,58]. For example, increased sialylation of membrane glycoproteins can engage inhibitory Siglec receptors on immune cells, dampening immune activation and contributing to immune tolerance or chronic infection [16]. Conversely, reduced sialylation or altered glycan composition can enhance leukocyte adhesion and infiltration, thereby promoting inflammatory tissue damage [16,45].

Autoimmune diseases further illustrate the pathological consequences of glycosylation dysregulation. Altered glycosylation of immune regulatory receptors and adhesion molecules can disrupt self-tolerance mechanisms and promote aberrant immune activation [16]. These findings highlight membrane protein glycosylation as a critical modulator of immune homeostasis and inflammatory signaling.

### 5.3. Metabolic and Cardiovascular Diseases

Emerging evidence links membrane protein glycosylation to metabolic and cardiovascular pathology. Membrane receptors and transporters involved in glucose uptake, lipid metabolism, and insulin signaling are subject to glycosylation-dependent regulation [6,21].

For example, altered glycosylation of insulin receptors and glucose transporters has been reported to influence receptor trafficking, ligand binding, and downstream signaling efficiency [6]. Such changes may contribute to impaired insulin sensitivity and metabolic dysfunction in conditions such as type 2 diabetes.

Within the vascular system, glycosylation of endothelial membrane proteins regulates cell–cell interactions, barrier integrity, and inflammatory signaling [7,70]. Dysregulated glycosylation of adhesion molecules and receptors on endothelial cells has been associated with endothelial dysfunction, a key early event in cardiovascular disease progression [71,72]. These findings indicate that membrane protein glycosylation links metabolic dysregulation to vascular pathology.

### 5.4. Disease Progression and Glycosylation Dynamics

A notable feature of disease-associated glycosylation changes is their dynamic nature. Glycosylation patterns can evolve during disease progression and in response to therapeutic interventions, reflecting ongoing cellular adaptation [6,8]. Recent advances in glycoproteomic and mass spectrometry-based approaches are enabling more detailed analysis of glycosylation dynamics over time and across disease states [25,26,27].

In cancer, for example, treatment-induced stress or targeted therapy can further remodel glycosylation pathways, altering glycan branching, terminal modifications, and receptor surface organization [15]. Such glycosylation remodeling may contribute to acquired drug resistance and tumor adaptation.

This temporal dimension highlights the importance of longitudinal analysis of membrane protein glycosylation, particularly in clinical contexts where disease monitoring and treatment response assessment are critical. Advances in glycoproteomics and glycan profiling technologies are beginning to enable such analyses at increasingly high resolution [25,26].

Despite substantial progress in characterizing disease-associated glycosylation changes, several limitations and open questions remain. In many cases, it is difficult to distinguish whether observed glycosylation alterations are causative drivers of disease or secondary consequences of broader cellular dysregulation. In addition, conflicting results across studies may arise from differences in analytical methods, sample heterogeneity, and context-specific regulation of glycosylation. Addressing these challenges will require more standardized approaches and careful integration of functional and clinical data.

## 6. Clinical Translation and Therapeutic Implications

The growing recognition of membrane protein glycosylation as an active regulator of disease biology has stimulated increasing interest in its clinical translation. Because membrane proteins are directly accessible to diagnostic probes and therapeutic agents, glycosylation-dependent modulation of their structure and function provides unique opportunities for biomarker development, therapeutic intervention, and personalized medicine [7,8,11]. In particular, disease-associated glycoforms of membrane proteins may offer clinically actionable information that complements genomic and proteomic biomarkers [15]. This section highlights emerging translational applications of membrane protein glycosylation and discusses key challenges that must be addressed to realize their clinical potential.

### 6.1. Glycosylation-Based Biomarkers and Diagnostics

Membrane protein glycosylation represents an attractive class of biomarkers because it integrates genetic, metabolic, and environmental signals into a single molecular readout [6,11]. In contrast to total protein abundance, which may remain relatively stable across disease states, glycosylation patterns frequently exhibit disease-specific alterations that enhance diagnostic and prognostic discrimination [7,8]. Compared with conventional protein-based biomarkers, glycosylation-based markers may provide additional information on functional state and disease-associated molecular alterations, potentially improving specificity and early detection [23,24].

In oncology, tumor-associated glycoforms of membrane proteins have been detected in tissue biopsies, circulating extracellular vesicles, and shed ectodomains, enabling minimally invasive diagnostic and prognostic assessment [69,73]. For example, aberrant glycosylation of membrane proteins such as MUC1 or PD-L1 has been associated with tumor progression and immune evasion [54]. These glycoforms frequently reflect aggressive disease phenotypes and therapeutic resistance.

Importantly, glycosylation-based biomarkers may capture dynamic changes during disease progression and treatment, providing information that complements static genomic markers [11,13]. Diagnostic approaches exploiting membrane protein glycosylation include glycan-specific antibodies, lectin-based assays, and mass spectrometry-based glycoproteomic profiling. The principles and translational pathways of these approaches are summarized in Figure 1d.

However, current analytical approaches still face several limitations. Glycoproteomic analyses are often challenged by incomplete coverage, limited sensitivity for low abundance glycoforms, and difficulties in resolving site-specific glycosylation with high confidence. In addition, the structural characterization of glycosylated membrane proteins remains technically demanding, particularly for large or heterogeneous complexes. These limitations can complicate data interpretation and may contribute to variability across studies, underscoring the need for improved analytical methods and standardized workflows.

### 6.2. Companion Diagnostics and Precision Medicine

As therapeutic targeting of membrane proteins becomes increasingly refined, the need for companion diagnostics that account for glycosylation-dependent variability has become apparent. Glycosylation can influence receptor conformation, ligand accessibility, and antibody binding, thereby contributing to heterogeneous therapeutic responses among patients [7,8]. Glycosylation-dependent variation in membrane proteins may also influence the performance of antibody-based diagnostics, as differences in glycan structure can affect epitope accessibility and detection sensitivity.

For antibody-based therapies, glycosylation of the target membrane protein can alter epitope exposure and binding affinity, affecting therapeutic efficacy [14,74]. For instance, glycosylation of immune checkpoint proteins such as PD-L1 can influence antibody recognition and immune checkpoint blockade responses [54]. Similarly, small-molecule inhibitors targeting membrane receptors may exhibit differential activity depending on glycosylation-dependent receptor clustering and signaling dynamics [21,66].

These observations highlight the importance of integrating glycosylation analysis into patient stratification strategies and precision medicine frameworks.

### 6.3. Therapeutic Targeting of Glycosylation Pathways

Beyond serving as biomarkers, glycosylation pathways themselves are emerging as actionable therapeutic targets. Because glycosylation regulates membrane protein conformation, clustering, and signaling dynamics, pharmacological modulation of glycosyltransferases, glycosidases, or nucleotide sugar metabolism offers a systems-level strategy to reprogram membrane protein function without directly altering genomic sequences [8]. This approach differs fundamentally from conventional receptor blockade strategies. While many of these approaches have shown promising results in preclinical models, their translation into clinical settings remains limited by challenges related to specificity, safety, and systemic effects. However, targeting glycosylation pathways may lead to pleiotropic effects, given the widespread roles of glycoproteins in diverse physiological processes. Strategies to mitigate these limitations may include targeted delivery approaches, such as antibody-based targeting or tissue-specific modulation of glycosylation pathways, to reduce off-target effects.

#### 6.3.1. Enzyme-Targeted Modulation of Glycosyltransferases

Aberrant expression or activity of specific glycosyltransferases is frequently observed in cancer and inflammatory diseases. For example, increased activity of the branching enzyme MGAT5 enhances N-glycan branching and promotes galectin-mediated receptor clustering, thereby sustaining growth factor receptor signaling [20,21].

Similarly, altered terminal sialylation mediated by sialyltransferases such as ST6GAL1 has been linked to immune evasion and metastatic potential [13]. Experimental suppression of sialyltransferase activity has demonstrated tumor growth inhibition and enhanced immune recognition in preclinical models [14]. Targeting fucosyltransferase pathways has also been proposed as a strategy to disrupt tumor–immune interactions and cell adhesion mechanisms [7,8].

However, enzyme-targeted approaches face intrinsic challenges. Glycosyltransferases often display overlapping substrate specificities, and systemic inhibition may affect multiple tissues due to the ubiquitous nature of glycosylation. Achieving disease-selective modulation without widespread disruption of physiological glycoproteins remains a central translational challenge [9,10].

#### 6.3.2. Targeting Nucleotide Sugar Metabolism

An alternative strategy involves modulation of nucleotide sugar biosynthesis pathways, particularly the hexosamine biosynthetic pathway, which supplies UDP-GlcNAc for N- and O-glycosylation. Increased metabolic flux through this pathway has been associated with cancer progression and metabolic disease [6,8].

Pharmacological modulation of nucleotide sugar availability can influence glycan branching and modify membrane receptor signaling outputs [6]. Such metabolic glyco-modulators act upstream of glycosyltransferases and may reshape multiple glycosylation-dependent signaling pathways simultaneously. However, the systemic nature of these pathways raises concerns regarding metabolic toxicity and unintended effects on normal tissues.

#### 6.3.3. Glycoengineering and Antibody-Based Strategies

In parallel with enzyme inhibition approaches, glycoengineering strategies have achieved significant clinical translation. Modification of Fc glycosylation on therapeutic antibodies can enhance antibody-dependent cellular cytotoxicity (ADCC) and modulate immune effector engagement [18].

Several clinically approved monoclonal antibodies incorporate Fc glycoengineering to improve therapeutic efficacy [18]. These successes demonstrate that glycosylation is not merely a structural feature but a tunable parameter in drug design. Extending glycoengineering approaches to membrane protein–targeted therapies, including bispecific antibodies and antibody–drug conjugates, represents an important emerging direction [15].

#### 6.3.4. Clinical Landscape and Combination Strategies

Despite strong preclinical evidence, clinical translation of glycosylation-targeted therapies remains in early stages. Most glycosyltransferase-directed agents are currently under preclinical development or early-phase investigation [8,13].

Future therapeutic strategies may benefit from combination approaches. Because glycosylation influences receptor clustering, immune recognition, and signaling persistence, combining glycosylation modulators with immune checkpoint inhibitors, kinase inhibitors, or metabolic therapies may enhance therapeutic efficacy (Table 7) [8,13].

## 7. Conclusions and Future Perspectives

Membrane protein glycosylation represents an important yet historically underappreciated layer of molecular regulation with broad implications for human disease. By influencing structural organization and signaling dynamics, glycosylation modulates the functional behavior of membrane proteins that occupy central roles in cellular communication and therapeutic intervention [7,21]. Increasing evidence indicates that glycan-mediated regulation can reshape receptor clustering, ligand accessibility, and signaling persistence at the cell surface, thereby linking molecular glycosylation states to cellular and clinical phenotypes [20,21]. Key challenges remain in achieving site-specific and functionally resolved characterization of membrane protein glycosylation, particularly in complex biological systems.

Despite growing interest in membrane protein glycosylation as a translational axis, several conceptual and practical challenges remain. Distinguishing causative glycosylation alterations from adaptive or secondary changes remains experimentally difficult, particularly in heterogeneous clinical samples [32,76]. In addition, tissue-specific glycosylation patterns and inter-individual variability complicate biomarker standardization and reproducibility across patient cohorts [77]. Systemic modulation of glycosylation pathways also raises concerns regarding off-target effects, given the ubiquitous presence of glycoproteins in normal physiology [9,10]. Addressing these challenges will require integration of advanced glycoproteomic techniques with structural biology, single-cell analysis, and systems-level approaches to better resolve glycosylation-dependent regulation.

This review has emphasized the value of a membrane protein-centric framework for understanding glycosylation, integrating biosynthetic mechanisms with structural effects, functional modulation, and disease relevance. Across cancer, immune, and metabolic disorders, disease-associated alterations in membrane protein glycosylation emerge as mechanistically meaningful features of disease biology rather than passive byproducts of cellular dysregulation [6,8].

From a translational perspective, this regulatory layer presents unique opportunities for biomarker discovery, therapeutic targeting, and precision medicine. Unlike static genetic alterations, glycosylation patterns are dynamic and potentially reversible, offering both diagnostic sensitivity and therapeutic flexibility [11,13]. Realizing this potential will depend on continued progress in glycoproteomic technologies, methodological standardization, and rigorous clinical validation [25,26].

Several priorities will likely shape the next phase of research in this field. First, systematic mapping of site-specific glycosylation on clinically relevant membrane proteins across disease stages will be essential. Second, integration of glycosylation data with functional and clinical outcomes will help distinguish causative mechanisms from correlative changes. Third, incorporation of glycosylation-aware strategies into drug development and clinical decision-making may refine existing therapeutic approaches and uncover new opportunities for intervention [32,78].

Membrane protein glycosylation represents an important regulatory layer linking molecular mechanisms to functional and clinical outcomes. Continued integration of mechanistic and translational studies will be essential for advancing this field.

## Figures and Tables

**Table 2:** Key regulatory steps in membrane protein glycosylation biosynthesis.

Biosynthetic Stage	Cellular Compartment	Regulatory Features	Disease Association	Key Reference
N-glycan attachment	Endoplasmic reticulum	OST activity, protein folding	Congenital disorders, loss-of-function	[5,6]
N-glycan remodeling	Golgi apparatus	Branching, terminal modifications	Cancer progression	[20,21]
O-glycosylation initiation	Golgi apparatus	GalNAc-transferase expression	Immune and inflammatory disease	[16,46]
Context-dependent regulation *	ER–Golgi network	Metabolic and signaling cues	Therapy resistance	[8,13]

* Context-dependent regulation refers to glycosylation-mediated effects that vary according to cell type, microenvironment, metabolic state, or disease context.

**Table 3:** Structural and biophysical consequences of membrane protein glycosylation with representative membrane protein examples.

Membrane Protein Class	Representative Proteins	Glycosylation Type	Structural/Biophysical Effect	Functional Consequences	Key Reference
Receptor tyrosine kinases	EGFR	N-linked	Stabilization of extracellular domains; regulation of receptor conformation	Ligand binding, receptor activation, signaling amplification	[20,21]
Immune checkpoint receptors	PD-L1	N-linked	Stabilization of surface expression; protection from degradation	Immune evasion, modulation of immune checkpoint signaling	[8,14]
Cell adhesion receptors	Integrins	N-linked and O-linked	Modulation of extracellular domain flexibility and receptor clustering	Cell–matrix adhesion, migration, mechanotransduction	[14]
Mucin-like membrane proteins	MUC1	O-linked	Extension of extracellular domain and formation of glycocalyx	Steric shielding, receptor accessibility, immune modulation	[45]
Ion channels	Voltage-gated sodium channels	N-linked	Regulation of folding, trafficking, and membrane localization	Channel gating, electrical signaling	[29]
Membrane transporters	GLUT1	N-linked	Stabilization of membrane localization and transporter turnover	Glucose transport and metabolic regulation	[6]
Immune receptors	T-cell receptor complex	N-linked	Glycan-mediated receptor organization via lectins	T-cell activation and immune signaling	[16,45]

**Table 4:** Glycosylation-associated therapeutic strategies targeting membrane proteins.

Target	Glycosylation Feature	Therapeutic Modality	Clinical Status	Representative Example	Key Reference
PD-L1	N-glycosylation stabilizes protein and affects antibody binding	Immune checkpoint inhibitor	FDA-approved	Anti-PD-1/PD-L1 antibodies	[54]
EGFR	N-glycan branching regulates receptor activation	Monoclonal antibody/TKI	FDA-approved	Cetuximab	[20,21]
HER2	Glycosylation affects receptor conformation and targeting	Antibody–drug conjugate	FDA-approved	Trastuzumab emtansine (T-DM1)	[15]
MUC1 (Tn/sialyl-Tn)	Aberrant O-glycosylation creates tumor-specific epitopes	Cancer vaccine/CAR-T	Clinical trials	MUC1-targeted therapies	[45,55]
CD19/CD22	Glycosylation influences antigen recognition	CAR-T-cell therapy	FDA-approved	CD19 CAR-T	[18]
Siglec ligands	Hypersialylation enables immune evasion	Glyco-immune checkpoint targeting	Preclinical/early clinical	Siglec-targeting agents	[55,56]

**Table 5:** Functional roles of membrane protein glycosylation across protein classes.

Protein Class	Glycosylation-Dependent Function	Pathological Impact	Key Reference
Receptors	Ligand binding, signaling duration	Cancer progression, therapy resistance	[8,20]
Adhesion molecules	Binding strength, mechanotransduction	Metastasis, inflammation	[7,14]
Transporters and channels	Trafficking, activity regulation	Metabolic disease, PK variability	[11,50]
Immune receptors	Activation thresholds, clustering	Autoimmunity, immune evasion	[16,56]

**Table 6:** Examples of disease-associated alterations in membrane protein glycosylation and their functional consequences.

Disease Context	Membrane Protein	Glycosylation Alteration	Enzymes/Pathways Involved	Functional Consequences	Key Reference
Cancer	EGFR	Increased N-glycan branching	MGAT5	Enhanced receptor clustering and sustained oncogenic signaling	[20,21]
Cancer	PD-L1	Stabilizing N-glycosylation	Glycosyltransferases; ER–Golgi processing	Stabilization of PD-L1 surface expression and immune evasion	[54]
Cancer	MUC1	Truncated O-glycans (Tn, sialyl-Tn antigens)	Dysregulated GalNAc-transferases	Reduced cell–cell adhesion; increased invasion	[45]
Cancer	Integrins	Altered sialylation and fucosylation	ST6GAL1, FUT8	Increased cell migration and metastatic potential	[14]
Immune disorders	Immune receptors (e.g., TCR complex)	Altered N-glycosylation	Glycosyltransferase regulation	Changes in receptor clustering and signaling thresholds	[16,45]
Chronic inflammation	Membrane glycoproteins	Increased sialylation	Sialyltransferases	Engagement of inhibitory Siglec receptors and immune suppression	[16]
Metabolic disease	Insulin receptor	Altered N-glycosylation	Hexosamine pathway flux	Impaired receptor signaling and insulin resistance	[6]
Cardiovascular disease	Endothelial adhesion molecules	Dysregulated glycosylation patterns	Multiple glycosyltransferases	Endothelial dysfunction and inflammatory signaling	[11]

**Table 7:** Clinical landscape of glycosylation-targeted therapeutic strategies.

Target Pathway	Representative Enzyme/Axis	Therapeutic Strategy	Clinical Stage	Disease Focus	Key Challenges	Key Reference
N-glycan branching	MGAT5 axis	Enzyme inhibition	Preclinical stage	Cancer	Redundancy, systemic glycoprotein effects	[21]
Sialylation	ST6GAL1, Siglec axis	Sialyltransferase inhibition	Early-phase clinical trials	Cancer, immune disorders	Immune modulation complexity	[13]
Fucosylation	FUT family	Fucosylation blockade	Preclinical stage	Oncology	Off-target glycoproteins	[8]
Nucleotide sugar metabolism	Hexosamine biosynthetic pathway	Metabolic modulation	Preclinical stage	Cancer, metabolic disease	Global metabolic impact	[6]
Fc glycoengineering	Fc glycan modification	Antibody optimization (ADCC enhancement)	Clinically approved	Oncology	Manufacturing consistency	[75]
Glycan–lectin interactions	Galectin–glycoprotein lattice	Disruption of receptor clustering	Preclinical/Discovery stage	Cancer	Target specificity	[20]

## Data Availability

No new data were created or analyzed in this study. Data sharing is not applicable to this article.

## Abbreviations

ADCC	Antibody-Dependent Cellular Cytotoxicity
Asn	Asparagine
ER	Endoplasmic Reticulum
ERAD	Endoplasmic Reticulum-Associated Degradation
Fc	Fragment crystallizable (region of antibody)
FUT	Fucosyltransferase
GALNTs	Polypeptide N-Acetylgalactosaminyltransferases
GPI	Glycosylphosphatidylinositol
GT	Glycosyltransferase
HBP	Hexosamine Biosynthetic Pathway
IgG	Immunoglobulin G
MGAT5	Mannosyl (Alpha-1,6-)-Glycoprotein Beta-1,6-N-Acetylglucosaminyltransferase
MS	Mass Spectrometry
OST	Oligosaccharyltransferase
PK–PD	Pharmacokinetics–Pharmacodynamics
Siglec	Sialic Acid-Binding Immunoglobulin-Like Lectin
ST6GAL1	Beta-Galactoside Alpha-2,6-Sialyltransferase 1
UDP-GlcNAc	Uridine Diphosphate N-Acetylglucosamine
UDP-Glucose	Uridine Diphosphate Glucose
